# Editorial of Special Issue “Magnesium in Human Health and Disease”

**DOI:** 10.3390/nu13082490

**Published:** 2021-07-21

**Authors:** Sara Castiglioni

**Affiliations:** Department of Biomedical and Clinical Sciences Luigi Sacco, Università di Milano, 20157 Milano, Italy; sara.castiglioni@unimi.it

The fundamental role of magnesium in human health is extensively discussed in the review by Fiorentini and colleagues [1]. Magnesium acts both as a signalling element and a metabolite in cell physiology, and its homeostasis is regulated by the balance between intestinal absorption and renal excretion. Hypomagnesemia is probably the most underestimated electrolyte imbalance in Western countries. Apart from being caused by an insufficient dietary magnesium intake, magnesium deficiency is frequently associated with obesity, type 2 diabetes and metabolic syndrome [2]. The review of Piuri and colleagues offers interesting insights into the biochemical derangements occurring when magnesium is deficient, and how these altered metabolic pathways increase the risk of developing metabolic syndrome and type 2 diabetes in obese individuals. The data analysed in the review of Găman and collaborators point to an association of magnesium concentrations in the body with dyslipidemia and related disorders [3]. Another study found a relationship between magnesium, the lipid profile and atherosclerosis in patients with kidney diseases [4]. In particular, the carotid intima media is thicker when triglycerides or LDL levels are high and magnesemia is low. On the contrary, when magnesium levels are high, this effect disappears [4].

A correlation between the reduction in both total and ionised circulating magnesium and chronic alcohol use disorder was also demonstrated by performing a meta-analysis [5]. In particular, inappropriately high magnesium excretion was reported in hypomagnesemic patients with chronic alcohol use disorder [5]. The major consequence of alcoholic or non-alcoholic liver disease is the accumulation of the extracellular matrix within the liver, leading to the development of cirrhosis. A study was conducted to analyse the association between magnesium and calcium intake and liver fibrosis. While no association was found between significant fibrosis and calcium intake, some findings suggest that high total magnesium may reduce the risk of fibrosis [6]. 

A retrospective cohort study conducted on kidney transplant patients demonstrated that a magnesium-deficient status, defined as serum magnesium < 0.7 mmol/L, increases the risk of urinary tract infections and viral infections in the first year after transplantation [7].

Hypomagnesemia is often found in cancer patients in association with therapy with cetuximab. This is due to the fact that cetuximab, interfering with Epidermal Growth Factor (EGF) signalling, downregulates magnesium intestinal influx, inhibiting the TRPM6 (Transient Receptor Potential Cation Channel Subfamily M Member 6) magnesium channel [8].

Apart from TRPM6, which is the key channel that mediates magnesium influx in intestinal cells, other transporters are also responsible for maintaining magnesium homeostasis, such as Transient Receptor Potential Cation Channel Subfamily M Member 7 (TRPM7), Magnesium Transporter 1 (MagT1) and Cyclin and CBS Domain Divalent Metal Cation Transport Mediator 4 (CNNM4). Analysis of these transporters using Genotype-Tissue Expression (GTEx) and The Cancer Genome Atlas (TCGA) revealed their overexpression in digestive cancers. In particular, a positive correlation between TRPM7/MagT1/CNNM4 expression was found in esophageal adenocarcinoma and pancreatic ductal adenocarcinoma, and a correlation between TRPM7 and MagT1 expression was found in colorectal cancer [9]. Since in pancreatic ductal adenocarcinoma, the correlation between TRPM7/MagT1/CNNM4 expression is associated with a low survival, these proteins could be proposed as new biomarkers to predict life expectancy.

It is known that magnesium is essential for musculoskeletal health. A study on mice which received a magnesium-deficient diet demonstrated that even a mild magnesium deficiency is sufficient to alter the expression of several genes critical for muscle energy metabolism, muscle regeneration, proteostasis, mitochondrial dynamics and excitation–contraction coupling [10]. Magnesium also has a fundamental role during myogenesis. Non-physiological magnesium concentrations induce oxidative stress in myoblasts, and this oxidative stress is responsible for the inhibition of myoblast membrane fusion, thus impairing myogenesis [11]. Magnesium also affects bone growth and remodelling. Magnesium depletion inhibits the cell cycle progression and proliferation of SaOS-2 osteosarcoma cells. Magnesium is mainly confined at the plasma membrane in quiescent cells, and, when cells are stimulated to grow, magnesium moves toward the inner areas of the cell. In contrast, when SaOS-2 cells are cultured in magnesium deficiency conditions, the ion remains confined at the plasma membrane, even when the cells are stimulated to grow. This condition is associated with the inhibition of SaOS-2 proliferation [12]. 

Magnesium also has an important role in the nervous system. In particular, high magnesium in cerebrospinal fluid appears to enhance the neural functions, while low magnesium induces neuronal diseases. Shindo and colleagues demonstrated that high concentrations of glutamate induce excitotoxicity because, after a transient increase in intracellular magnesium due to its release from mitochondria, the cytosol magnesium concentration dramatically decreases. The authors showed that, by inhibiting the magnesium extrusion under toxic concentrations of glutamate, the excitotoxicity induced by glutamate is reduced, thus pointing out the importance of magnesium in the regulation of neuronal survival [13].

Magnesium deficiency is associated with mild and moderate tension-type headaches and migraines. A number of double-blind, randomised, placebo-controlled trials have demonstrated that magnesium supplementation is efficacious in relieving headaches. In particular, magnesium pidolate may have high bioavailability and good penetration at the intracellular level [14].

A large body of literature suggests a correlation between magnesium deficiency and stress. In particular, stress could induce magnesium deficiency, and, in turn, low magnesium levels could increase the susceptibility to stress, resulting in a magnesium and stress vicious circle [15].

Magnesium has also been proposed as therapy for hypertension. Rosanoff’s review of 49 clinical trials shows that magnesium oral administration in association with treatment regimens in patients with partially controlled hypertension may be promising to control the blood pressure without increasing antihypertensive drugs [16].

Despite the fact that magnesium deficiency is very frequent in the population and is associated with altered levels of other electrolytes and various diseases, its diagnosis still represents a challenge because, to date, there is no gold standard to measure its concentration. The study of Zhan and colleagues showed that to assess magnesium bioavailability, circulating ionised magnesium might be a more sensitive measure of acute oral intake of magnesium than serum and urinary magnesium [17]. Orlova and co-workers proposed a magnesium deficiency questionnaire based on questions grouped into five categories: wellbeing, lifestyle, pregnancy, disease and medication, as a non-invasive assessment tool that could help to diagnose magnesium deficiency [18].

It is known that magnesium, particularly magnesium oxide, is clinically prescribed as a laxative. Compared to the newer drugs to treat constipation, magnesium is safe and of low cost, but daily use, particularly in patients with renal impairment, might lead to hypermagnesemia. Therefore, monitoring the magnesium concentration should be recommended [19].

Hypermagnesemia is an uncommon problem that can be caused by acute or chronic kidney diseases, hypothyroidism or corticoadrenal insufficiency and impacts on cell, tissue and organ functions, leading to many disorders. An analysis of serum magnesium concentrations in hospitalised patients revealed that either hypo- or hypermagnesemia was associated with an increased risk of in-hospital mortality, with dysmagnesemia being associated with severe diseases [20].

Overall, the 20 papers published in this Special Issue highlight that an adequate magnesium dietary intake, and maintenance of a correct magnesium homeostasis are essential for human health. To control magnesium availability, it would be advisable, in daily clinical practice, to include magnesium in the evaluation of blood ionograms.
